# How should we think about the unprecedented weight loss efficacy of incretin-mimetic drugs?

**DOI:** 10.1172/JCI174597

**Published:** 2023-10-02

**Authors:** Yang Gou, Michael W. Schwartz

**Affiliations:** University of Washington Medicine Diabetes Institute, Department of Medicine, University of Washington, Seattle, Washington, USA.

## Introduction

The introduction of highly potent incretin mimetic drugs has ushered in a new era of obesity and type 2 diabetes (T2D) treatment. Newer versions of these drugs produce weight loss comparable to what previously could be achieved only by bariatric surgery, and similarly impressive antidiabetic effects are elicited in patients with T2D. It is unsurprising, therefore, that this has become a very competitive area of pharmaceutical development, with new compounds being introduced at an impressive pace. Yet our understanding of how these drugs work is far from complete.

The term incretin refers to peptide hormones secreted by intestinal enteroendocrine cells in response to nutrient ingestion that increase the amount of insulin secreted by pancreatic beta cells in response to a glucose challenge. Some incretins act directly on beta cells while others mediate their effects indirectly, but the overall effect is important — failure to mount the normal incretin response to a meal impairs glucose tolerance by reducing insulin secretion.

## From physiology to pharmacotherapy

The two most physiologically relevant incretins are glucagon-like peptide-1 (GLP-1) and glucose-dependent insulinotropic peptide (GIP). These peptides mediate their effects by binding to GLP-1 and GIP receptors, respectively, and their ability to augment insulin secretion drew the attention of the pharmaceutical industry decades ago. In 2005, the first long-acting GLP-1 receptor agonist compound (exenatide) was approved for the treatment of T2D by the Food and Drug Administration (FDA), and a total of 5 GLP1-receptor agonist agents are approved in the US as of 2023 (lixisenatide, liraglutide, dulaglutide, two versions of exenatide and 3 versions of semaglutide).

Based on preclinical evidence that GLP-1 and GIP promote insulin secretion via distinct mechanisms, pharmaceutical companies next sought to develop novel dual agonist incretin mimetics. In 2022, tirzepatide, which incorporates agonists of both GIP and GLP-1 receptors into a single molecule, was approved by the FDA for T2D. The antiobesity and glucose-lowering effects of this drug appear to outperform all other currently available medications for T2D (1). Earlier this year, the *New England Journal of Medicine* published the results of a phase II trial of a triple hormone agonist (Retatrutide), a long-acting drug that combines a potent glucagon receptor agonist with agonists of both GLP-1 and GIP receptors into a single molecule (2). This early phase trial demonstrated unprecedented weight loss efficacy in obese humans (–24.2% weight reduction after 48 weeks of treatment on the highest dose).

Originally developed solely as insulin secretagogues, the brain appears to be the more important target for the effects of incretin mimetic drugs on weight loss. Of particular relevance to the action of incretin mimetics is a complex set of physiological responses set in motion by food ingestion, collectively referred to as gut-brain signaling (3).

## Gut-brain signaling: A key target for the action of incretin mimetics

As nutrients are consumed, gut-brain signals are transmitted by both neural and humoral mechanisms. As the name implies, these signals engage brain systems that promote homeostasis in response to the metabolic challenge posed by nutrient absorption into the blood stream. Nutrient consumption triggers secretion of a considerable number of peptide hormones by intestinal enteroendocrine cells, only some of which function as incretins (4). Ingested nutrients also activate afferent vagal and somatosensory nerves that innervate the gastrointestinal (GI) tract. Beyond the effect of food consumption to augment insulin secretion, gut-brain signals are responsible for the satiating effect of food ingestion that leads to meal termination (5).

Specifically, the sense of fullness following a meal is mediated by both afferent neural signals and hormones arising from the GI tract (such as GLP-1 and cholecystokinin (CCK)). These humoral and neural signals converge on neurons located in hindbrain areas such as the nucleus of the solitary tract and area postrema, activation of which inhibits feeding (among many other effects) (5). The release of incretin peptides can therefore be viewed as just one of many GI responses to nutrient ingestion that impact metabolism, autonomic function, and behavior, and increased insulin secretion is just one of many effects elicited by incretin peptides.

By potently engaging this gut-brain signaling pathway, incretin peptides at pharmacological doses cause weight loss by inducing a strong sense of satiety that can cross over into nausea and overt disdain at the prospect of eating (6). In addition to the potency with which they bind to and activate their respective receptor(s), the efficacy of incretin mimetics depends on a very long duration of action, as a short-lived drug effect would allow hunger to rapidly return and lost weight to be regained. The overarching goal of incretin mimetic drug development is therefore the continuous, potent pharmacological activation of incretin receptors, resulting in unrelenting appetite suppression and dramatic, continuous weight loss. Based on data from human subjects who were studied after 20 weeks of treatment with either semaglutide or placebo, this goal is clearly achievable (7). Despite having lost 10% of their body weight, the daily calorie intake of individuals receiving semaglutide was reduced by approximately a third relative to controls, and subjective rating scores revealed markedly reduced hunger, increased perception of fullness and/or satiety, and decreased interest in food (7). What is particularly striking about this study is that these subjective experiences are precisely the opposite of the normal response to voluntary weight loss. Indeed, adaptive responses to weight loss — comprising both increased food intake and decreased energy expenditure — constitute the single biggest obstacle to successful, long-term obesity treatment (8).

## Energy homeostasis, AgRP neurons, and the adaptive response to weight loss

**Figure 1:**
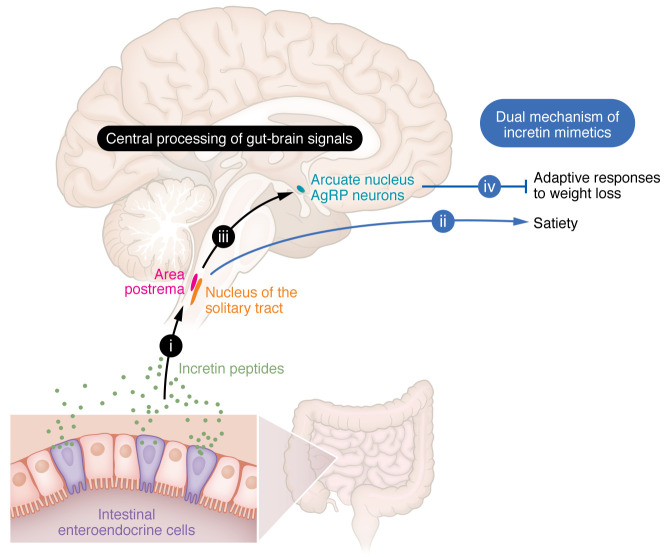
Dual mechanism of incretin mimetics. Gut-brain signaling is activated by incretin peptides released from enteroendocrine cells during a meal (i). By activating neurons in the hindbrain nucleus of the solitary tract and area postrema, these signals induce the perception of satiety and hence reduce food intake (ii). Lying downstream in this pathway are AgRP neurons in the hypothalamic arcuate nucleus, powerful drivers of the adaptive responses to weight loss, and these neurons are inhibited by incretin-induced activation of gut-brain signaling (iii). Thus, the potent weight loss efficacy of incretin mimetics is mediated not only by a sustained reduction of food intake (via increased satiety) but by blunting the normal adaptive responses to weight loss (iv).

As these adaptive responses are typically mounted in response to weight loss of approximately 5% or more, irrespective of one’s starting weight, it makes sense to develop weight loss therapeutics that block this response (8). Based on evidence discussed above, it can be argued that semaglutide indeed blocks the normal feeding response to weight loss (Figure 1). Whether semaglutide also blunts the adaptive decrease of energy expenditure that normally accompanies weight loss requires additional study. A key point here is that targeting gut-brain signaling to induce satiety and blocking adaptive responses to weight loss constitute two distinct mechanisms of action.

Meal-to-meal control of food intake by gut-brain signals can be viewed as a component of a larger system that promotes stability in the amount of body fuel stored as fat. This energy homeostasis is achieved by correcting mismatches between energy intake and energy expenditure over long time intervals. On a day-to-day basis, mismatches of energy balance are inevitable — there is no way to effectively match calories consumed to energy expended over the short term. But when a mismatch is sufficient to change body fat mass, the effect is detected in the brain by changes in the circulating levels of leptin and other adiposity negative feedback signals that vary with the level of body fat (9).

The neurocircuitry that responds to this afferent input is complex and distributed across many brain areas, but neurons in the hypothalamic arcuate nucleus (ARC) play a key role (9). For example, neurons that express agouti-related peptide (AgRP) are activated by weight loss and are potent drivers of feeding behavior (10, 11); in animal models, the effect of weight loss to trigger an adaptive increase of food intake is prevented if these neurons are silenced or ablated (12). As these neurons also express neuropeptide Y (NPY) and GABA, food intake is stimulated by multiple, complementary mechanisms following their activation. Thus, whereas AgRP promotes hyperphagia and weight gain by inhibiting a key brain system for body weight control, activation of NPY receptors lying downstream of AgRP neurons can also potently stimulate feeding (10). At the same time, GABA projections from AgRP neurons to brain areas such as the parabrachial nucleus block aversive responses to noxious stimuli (including GI distress) that might otherwise suppress feeding (13). But AgRP neurons are not the only neurons that promote recovery of lost weight — they are better viewed as a key node within a highly integrated and complex neurocircuitry that defends against weight loss.

While data on the role played by AgRP neurons in the adaptive response to weight loss derives largely from preclinical studies, the melanocortin system, which is inhibited by AgRP, is also integral to energy homeostasis in humans. This assertion is based on abundant evidence that, just as in rodent models, human obesity is induced by mutations of genes encoding key melanocortin system components: the melanocortin 4 receptor, pro-opiomelanocortin, and several other required proteins (9). In addition, AgRP neurons are inhibited by leptin, and leptin-deficient mice and humans both exhibit a severe obesity phenotype (10). To a considerable degree, therefore, homology in the energy homeostasis system exists across mammalian species.

## Two distinct mechanisms underlying weight loss induced by incretin mimetics

How then do incretin mimetics cause weight loss well in excess of the 5% threshold without seeming to activate adaptive responses to weight loss? We consider here 3 possibilities, that incretin mimetics (a) overwhelm adaptive responses to weight loss, such that they don’t matter; (b) reset the body weight set point, such that adaptive responses are mounted at a much lower body weight threshold; or (c) actively inhibit the adaptive response, such that it is prevented from occurring. While additional work is needed to sort through these possibilities, existing evidence favors the latter. Namely, emerging data suggest that AgRP neurons are inhibited by most gut-brain signals (14, 15), including GLP-1 receptor agonists (16). Incretin mimetic drugs therefore appear to impair the adaptive response to weight loss, at least in part, by inhibiting AgRP neurons.

From these considerations, we surmise that the unprecedented efficacy of incretin mimetic drugs involves two distinct actions — potent and sustained activation of gut-brain signaling to continuously suppress appetite and, as weight is lost, prevention of adaptive responses that promote weight regain (Figure 1). These mechanisms of action are important in considering what happens when a formerly obese individual discontinues one of these drugs after experiencing pronounced weight loss (e.g., –20% reduction of body weight): the lost weight is regained at an extraordinary pace (17) — even faster than it was lost — presumably because the energy homeostasis system, having awakened after being suppressed for months, responds vigorously to the detection of body fat mass well below its biologically defended level. Preventing this type of response requires greater insight into how the energy homeostasis system works and how it is impacted by these drugs.

A second concern pertains to prospect of exposing patients to potent and continuous activation of two or more different hormone receptors, each distributed widely throughout the body, potentially for a period of many years. How confident should we be that such an approach will not have unanticipated consequences? Clinical safety trials involving thousands of subjects over a period of months may not suffice to detect problems that surface only after millions of individuals have been treated for years.

Finally, a more complete understanding of critical nodes at the interface of gut-brain and energy homeostasis systems may enable the identification of drug targets that can achieve outcomes comparable to those of current incretin mimetic drugs — but in a far more specific manner and hence with a lower potential for adverse effects with long-term use.

## Concluding remarks

Until very recently, the identification of compounds with efficacy comparable to that of bariatric surgery was considered the holy grail of obesity drug development. Now that this goal has been achieved, perhaps we can set our sights on the specific neurocircuits responsible for the defense of elevated body weight and glycemia in patients with obesity and T2D. Therapeutic targeting of specific neuronal subsets within these circuits, rather than multiple hormone receptors distributed throughout the body, offers a far more specific approach to the treatment of obesity-associated metabolic disease.

## Version 1 10/02/2023

Electronic publication
